# Anatomical Parcellations of Brodmann's Areas 4 and 6: A Study on Cortical Thickness for Improved Neurosurgical Planning

**DOI:** 10.7759/cureus.41280

**Published:** 2023-07-02

**Authors:** Albert F Alan, Michelle Ennabe, Bambi Wessel, Bryan T Klassen, Kai Miller

**Affiliations:** 1 Neurological Surgery, Mayo Clinic, Rochester, USA; 2 Neurology, Mayo Clinic, Rochester, USA

**Keywords:** primary somatosensory cortex, layer v, betz cell, parcellations, divisions, supplementary motor cortex, primary motor cortex, area 6, area 4, korbinian brodmann

## Abstract

The cerebral cortex, comprising six layers known as the neocortex, is a sheet of neural tissue that contains regions for neurosurgical planning, including the primary motor cortex (PMC), the supplementary motor cortex (SMA), and the primary somatosensory cortex (PSC). However, knowledge gaps persist concerning the transition points between areas 3 to 4 and 4 to 6 and the SMA's extent. This study aims to develop a non-invasive protocol using T1/T2 weighted imaging to identify crucial anatomic borders around the primary and supplementary motor cortex for neurosurgical planning. A comprehensive literature search on the cytoarchitectonic borders of Brodmann's areas 3a, 4, and 6 was conducted, and relevant articles were selected based on their examination of these borders. The primary motor cortex was found to be the thickest region in the human brain, with discernible differences in thickness between areas 4 and 6. T2-weighted images revealed significant cortical thickness differences between the precentral and postcentral gyrus. Various methods have been employed to parcellate borders between cortical regions, including Laplace's equation and equi-volume models. A triple-layer appearance in the primary motor cortex and a novel method based on myelin content demonstrated consistent agreements with historically defined cytoarchitectonic borders. However, differentiating areas 4 and 6 from MR imaging remains challenging. Recent studies suggest potential methods for pre-surgically identifying the primary motor cortex and examining differences in cortical thickness in diseases. A protocol should be established to guide neurosurgeons in accurately identifying areas 4 and 6, possibly using imaging modalities superimposed on myelin maps for differentiation and determining area 6's anterior extent.

## Introduction and background

Approximately 75% of the cerebral cortex is made up of pyramidal cells. The cerebral cortex comprises six layers called the neocortex. The six layers from superficial to deep are molecular or plexiform (layer I), external granular (layer II), external pyramidal (layer III), internal granular (layer IV), internal pyramidal (layer V), and multiform or fusiform (layer VI). [Brodmann and Gary] The focus of this paper will pay to be on the parcellations of the primary motor cortex (PMC), the supplementary motor cortex (SMA), and the primary somatosensory cortex (PSC). The primary motor cortex (area 4) comprises prominent layers III and V, with giant pyramidal cells located in the latter. These Betz cells are generally considered exclusive to area 4. As a result, they are not typically seen in the supplementary motor cortex (area 6) or primary somatosensory cortex (areas 1, 2, 3a, and 3b). Brodmann’s cytoarchitectural analysis found the internal granular layer absent in the PMC and SMA but not in the PSC. Immediately following Brodmann’s discovery, Von Economo and Koskinas classified the neocortex into five parts: agranular, frontal, parietal, polar, and granular [1]. Based on these classifications, the PMC was considered part of the agranular cortex. At the same time, the PSC is defined as the granular cortex based on the presence of the internal granular layer. 

Throughout many advances in the health sciences, precision cortical mapping has not changed since the discovery of the motor homunculus by Penfield and Boldrey [2]. Likewise, optical imaging by Haglund et al. became the gold standard for localizing language and the sensory-motor cortex during neurosurgical operations [3]. These techniques have served neurosurgeons during infiltrative tumor resections near eloquent brain regions. However, cortical mapping is restricted to patients undergoing open brain surgery [3,4]. After all these advancements, gaps still exist within the transition points of areas 3 to 4, and 4 to 6, respectively. Furthermore, there is a lack of information in the literature quantifying how far the supplementary motor cortex extends in the rostral direction. Establishing a non-invasive protocol utilizing T1-weighted/T2-weighted imaging can provide useful information for neurosurgical planning in areas 3, 4, and 6. Moreover, non-invasive cortical mapping could provide information about important anatomic borders surrounding the primary and supplementary motor cortex.

## Review

Methods

A comprehensive literature search was conducted using Google Scholar, Embase/Medline, PubMed, SCOPUS, and Cochrane databases. The searches were carried out from the inception of each database up to January 2023. The first search used the terms "human", "cortical thickness", "Brodmann", "area 4", "area 6", "motor", "myelin", and "thick" and was analyzed in combination. To broaden the search criteria, a second search was conducted using the terms "human", "cortical thickness", "Brodmann", "area 4", "area 6", "thick", and "motor", which were also analyzed together. Relevant articles were selected based on their investigation of the cytoarchitectonic borders of Brodmann's areas 3a, 4, and 6. Additionally, the references section of key papers was analyzed for further understanding of the parcellations of Brodmann's areas 3/4 and 4/6. 

Results

**Figure 1:**
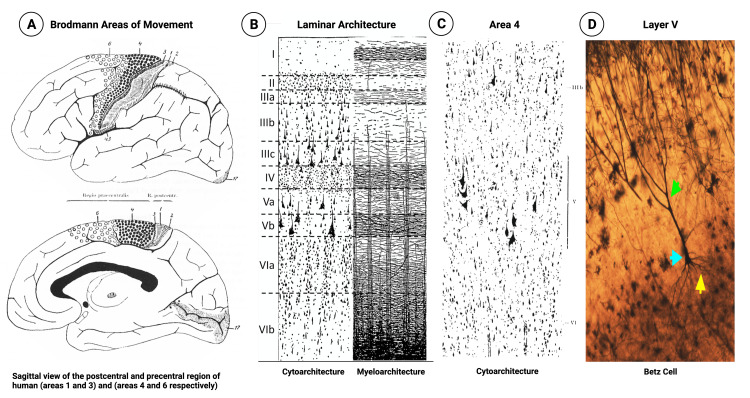
Neuroanatomy illustrations: sagittal views, cortical composition, and Betz cells of the primary motor cortex A: Sagittal view areas 1, 3, 4, and 6. B: cytoarchitectonic and myeloarchitectonic composition of the cerebral cortex. C: zoomed-in version of Brodmann's area 4 of the primary motor cortex, illustrating the Betz cell in layer V. D: Betz cell of plains zebra Betz. Light Blue Arrow: cell body | Yellow Arrow: dendritic arborization | Green Arrow: Axonic Betz cells Projections (which ultimately feed into) → Corona Radiata → Internal Capsule → Midbrain → Pons → Medulla → Spinal Cord. Figures 1A, 1B, and 1C: taken with permission from Dr. Nicola Palomero-Gallagher's original publication to be reproduced and derived from Brodmann, K. (1914). Physiologie des Gehirns. In von Bruns, P. (ed.), Neue Deutsche Chirurgie (pp. 85–426). Stutgart: Verlag von Ferdinand Enke. Figure 1D: Dr. Bob Jacobs, the creator of the image, has granted permission to use this image in the present article.

In 1909, Korbinian Brodmann parcellated 52 separate regions in the cerebral cortex of small and large mammals [5,6]. Brodmann analyzed standardized histological sections of brain autopsies and found similarities regarding the cellular arrangement, size, density, and cortical thickness across all the species [5,6]. An example of this can be found in Figure 1. Without any advanced computer software, the German neurologist was able to isolate the primary motor cortex as the thickest region in the human brain. When comparing area 4 to area 6, Brodmann discovered the thickness to be 3.0-4.5mm and 3.8-3.8mm, respectively. Exactly 112 years later, scientists are struggling to parcellate the differences in thickness between these two areas during clinical brain mapping applications [7]. Within the cytoarchitecture of layer I in areas 4 and 6, Brodmann found the thickness to be .35mm and .37mm, respectively [5]. Further examination of the cytoarchitecture found that areas 4 and 6 lacked an inner granular layer. Likewise, area 4 can be further distinguished from area 6 by the presence of Betz giant cells, with the outer cellular layers denser than the inner [8]. Moreover, layer III only constitutes 40%-45%, while layer III and layer V combined make up 70% of the cortical thickness of area 4 [8]. However, as Brodmann began to follow the rostral border of the primary motor cortex, he noted a purely subjective gradual transition of Betz cells to area 6 [5,9]. Likewise, within area 4, the cellular density of Betz giant cells decreases from medial to lateral as well as ventrally from the central sulcus [5,8]. In like manner, the total cortical thickness decreases ventrally from area 4 to area 6, with the latter changing in anatomic structure in the dorsoventral direction [5,6].

Dinse and Preim further analyzed the histological myeloarchitectonic sections of the primary motor-somatosensory region M1/S1 (primary motor cortex/ primary somatosensory cortex) [8]. They found that area 4 showed increased myelination between layers II and V when compared to areas 3a, 3b, 1, and 2. However, the same issues remain as sections were taken from postmodern samples. Thus, quantitative cortical layering modeling was required to parcellate the borders between M1/S1 during clinical brain mapping applications. Previous studies utilized Laplace’s equation, which took advantage of the geometric structure seen in the anterior and posterior bank of the central sulcus as a harmonic function [10]. The ratio of the anterior to posterior banks for each hemisphere was 1.50, supporting previous MR techniques focusing on the central sulcus [10,11]. The authors contemplated using Laplace and equidistant models; however, none of the approaches explicitly modeled the volume-preserving criterion [12]. Thus, the authors used the equi-volume model, as validated by Waehnert et al., to qualitatively parcellate the ex-vivo postcentral gyrus as well as follow the architecture in the cerebral cortex [8]. With this application, Dinse and Preim were able to show a lower T1 value in area 4 when compared to area 3 in an in vivo model [8]. Thus, the cortical thickness of the primary motor cortex is greater than that of the somatosensory cortex. Likewise, according to Brodmann, von Economo, and Koskinas, area 1 has similar regions to the premotor cortex in area 6 [1,5]. 

In the presence of vasogenic edema, Biega et al. used T2-weighted images to parcellate the anterior and posterior banks of the central sulcus [13]. The authors identified 13 patients with brain tumors near the central sulcus suffering from vasogenic edema. T2-weighted measurements were analyzed from neighboring areas, including the frontal and parietal lobes. They found a twofold increase in cortical thickness between the anterior and posterior banks of the central sulcus, despite the mass effect seen from the brain tumor. When comparing T1-weighted and T2-weighted images, it was clear that the latter demonstrated superior visualization of the cortical thickness between the precentral and postcentral gyrus. T1-weighted images were obscured due to the mass effect resulting from the brain tumor-induced vasogenic edema. The average cortical thickness of the precentral gyrus was 3.34 mm, compared to the postcentral gyrus, which measured 1.80mm. The author’s results correspond with the historic cytoarchitectonic differences between the motor and sensory cortex. 

To help distinguish area 4 from areas 3a, 3b, 2, and 1. Kim et al. used thin-section axial double inversion recovery brain magnetic resonance to illuminate a triple-layer appearance in the primary motor cortex [14]. The authors studied 191 normal patients (5-76 years old) with 3.0-T MR imaging. Likewise, ten patients with brain tumors underwent cortical mapping for validation of the triple-layer appearance of the primary motor cortex. It was concluded that the appearance of a triple layer was consistently present in the primary motor cortex but not in the somatosensory cortex. The authors found that the triple-layer structure was not observable in patients under 10 years of age. They concluded this was due to underdeveloped age-related myelination in these younger patients. 

As advancements in technology commenced, Glasser and Essen presented a new method of mapping cortical areas based on their myelin content [15]. This was illustrated by T1-weighted (T1w) and T2-weighted (T2w) MRI 3T pulse sequences utilizing a T1w/T2w ratio to enhance the contrast-to-noise ratio for myelin. The authors found consistent agreements between the myelin maps and historic cytoarchitectonically-defined borders dating back to 1909. Axial sections comparing the anterior and posterior banks of the central sulcus found that areas 4 and 3b were more myelinated than areas 6, 3a, and 1 [15]. Glasser and Essen confirmed that area 4 was increasingly more myelinated than area 6 and its divisions [15]. The authors found a heavily myelinated caudal to the rostral gradient in area 6, which gradually becomes less heavily myelinated from the SMA to pre-SMA transition. Glasser and Essen’s findings are consistent with the caudal to rostral decrease in myelination in area 4 and area 6 from Hopf 1956. Corticospinal projections from area 4 are consistent with a heavier myelination gradient corresponding to the somatotopic organization of the distance traveled from the lower body when compared to the upper body [15].

Discussion

**Figure 2:**
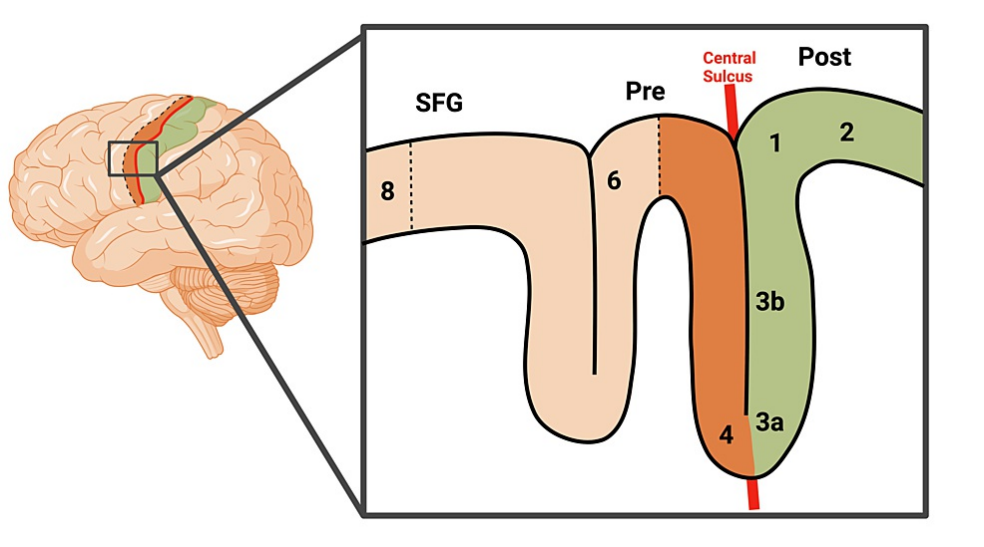
Variability in the transition points between Brodmann's areas 4/6 and 6/8 The transition from Brodmann's area 3a/4 to the primary motor cortex's area 4 by means of a notable thickness increment. The demarcation or demarcating frontier between regions 4 and 6 has been observed to exhibit substantial heterogeneity across individuals, indicating that it may vary from person to person. This inter-individual variability is evident in the dashed line depicted in the aforementioned figure, which represents the boundary between areas 4 and 6. Furthermore, there is considerable variability in the transition between areas 6/8 located anterior to the pre-central sulcus. Created with BioRender.com

Since its first introduction over 11 decades ago, Korbinian Brodmann has laid the cytoarchitectonic foundation by defining 52 separate areas of the cerebral cortex. The implications of Brodmann’s discovery of areas 4 and 6 helped establish the somatotopic discovery of the motor homunculus by Dr. Penfield. However, even Brodmann himself found the transition from areas 4 to 6 to be gradual and subjective [5,9]. Despite over 100 years of technological advancements, scientists are still struggling to parcellate areas 4 and 6 from MR imaging. Based on our current understanding, Figure 2 depicts the schematic of parcellation at the transitional junctures between Brodmann's areas 4/6 and 6/8. What we do know is that the cortical thickness of the precentral gyrus is much thicker than that of the post. Furthermore, Brodmann’s identification of the cellular density of Betz cells in layer V of area 4 gradually decreasing from medial to lateral corresponds to Dr. Penfield’s homunculus.

The work of Kim et al. has helped identify yet another differentiating factor that distinguishes the motor and somatosensory cortex [14]. The utilization of a double inversion recovery brain magnetic resonance imaging sequence revealed a triple-layer appearance in the primary motor cortex and not in the somatosensory cortex. These findings were found to be independent of age or sex in patients older than 10 years. During the first to third decade of life, we can observe a notable increase in myelin synthesis. This process results in an underdeveloped clear triple-layer configuration within the primary motor cortex in individuals under 10 years old. This development helps explain why children younger than 10 years have not yet accumulated a substantial amount of myelin. Furthermore, the authors only reviewed the double inversion recovery imaging in selected brain regions. Thus, it may be possible that the triple-layer appearance at 3.0T is not exclusive to the primary motor cortex. Similarly, it is imperative to employ a more extensive cohort in order to substantiate whether additional cerebral pathologies characterized by significant mass effect and edema have an impact on the uniformity of the triple-layer visual pattern observed within Brodmann's area 4. 

All things considered, the implications of these findings could help the identification of the primary motor cortex in the pre-surgical setting. Additionally, it may help clinicians analyze the differences in cortical thickness within diseases such as amyotrophic lateral sclerosis, primary lateral sclerosis, or corticobasal degeneration [14]. 

The central sulcus holds significant clinical importance, and to study its anatomical divisions without invasive procedures, various non-invasive methods have been utilized, including functional MR imaging and positron-emission tomography, to parcellate the sulcal anatomy [13]. However, even these methods are not 100% accurate in identifying the central sulcus. Thus, Biega et al. used T2-weighted imaging modalities to measure cortical thickness between the anterior and posterior bank of the central sulcus. The implication of these results can help neurosurgeons preoperatively localize the primary motor cortex in preparation for brain tumor resection, especially during settings of vasogenic edema obscuring normal landmarks that would normally localize the central sulcus. Unfortunately, the authors did not go further into identifying the parcellation of areas 4 and 6. Their results do not illustrate where area 4 transitions into area 6. Furthermore, the imaging modalities used do not take into account how far anterior area 6 extends. Glasser and Essen take note of these issues and provide a method to differentiate areas 4 from 6. By dividing the methods seen in Biega et al., Glasser and Essen utilized a T1w/T2w ratio to enhance the contrast to noise ratio for myelin. Above all, they illustrated the primary motor cortex to be more heavily myelinated than the supplementary motor cortex. No other article was found that directly answers this question. Unfortunately, the authors have not provided a clear transitional demarcation of the anterior extent of area 6. Due to the inter-variability seen in area 6 from individual to individual, no preset landmark can be established.

## Conclusions

It is recommended that a protocol is established to guide neurosurgeons in properly identifying areas 4 and 6. Protocols that take advantage of the cerebral cortex geometry could find a gradual transition out of the supplementary motor cortex based off cortical thickness. Likewise, Brodmann, von Economo, and Koskinas found similarities between areas 1 and 6. Thus, imaging modalities superimposed on myelin maps not only can help parcellate areas 4 from 6 but can also answer how far anterior area 6 extends to. The implications of these results could advance neurosurgical planning and detection of motor pathologies in a noninvasive manner.
